# T149. METACOGNITIVE DEFICITS IN INTEROCEPTION ARE ASSOCIATED WITH DISSOCIATIVE EXPERIENCES IN PATIENTS WITH FIRST EPISODE PSYCHOSIS

**DOI:** 10.1093/schbul/sby016.425

**Published:** 2018-04-01

**Authors:** Sarah Garfinkel, Kathy Greenwood, Charlotte Rae, Geoff Davies, Cassandra Gould van Praag, Anil Seth, Medford Nick, Hugo Critchley

**Affiliations:** University of Sussex

## Abstract

**Background:**

Dissociative experiences, including depersonalization and derealisation, represent perturbations of consciousness and selfhood, and are commonly reported by patients during early stages of a psychotic illness. The continuity and integrity of a conscious sense of self is proposed to be grounded upon the control of internal physiological state and its predictive representation through interoception, i.e. the sensing of internal bodily changes. We tested the hypothesized relationship between dissociation and interoceptive deficits in patients with first episode psychosis (FEP), combining behavioural testing with functional neuroimaging.

**Methods:**

Individuals with first episode psychosis (N=41) and matched community control participants (N=21) performed an interoceptive task (heart-tone synchrony judgments) during functional magnetic resonance imaging (fMRI). Trial-by-trial confidence ratings indexed subjective performance, and measures of metacognitive interoceptive awareness (insight) were derived from confidence-accuracy correspondence. We tested for regional brain activity relating to dissociative symptom scores and objective, subjective and metacognitive aspects of interoception.

**Results:**

In patients with FEP, metacognitive impairments in interoception predicted magnitude of dissociative symptoms, accompanied by hypoactivation of right insula cortex. Other dimensions of interoception, and accuracy, confidence and metacognitive insight on an exteroceptive task were unrelated to dissociative symptoms and there were no group differences between FEP patient and control groups.

**Discussion:**

Our findings suggest that symptoms of disturbed conscious integrity and selfhood in early psychosis arise through selective disruption of higher-order metacognitive representations of interoceptive signals. Brain systems supporting the conscious integration of bodily feelings may represent a target for interventions to enhance functioning and, speculatively, mitigate illness progression in psychosis.

